# Relevance of gap junctions and large pore channels in traumatic brain injury

**DOI:** 10.3389/fphys.2014.00031

**Published:** 2014-02-11

**Authors:** Nora Prochnow

**Affiliations:** Department of Neuroanatomy and Molecular Brain Research, Ruhr-University BochumBochum, Germany

**Keywords:** traumatic brain injury, Connexin, Pannexin, penumbra, glial scar, astrocytes, blast, excitotoxicity

## Abstract

In case of traumatic brain injury (TBI), occurrence of central nervous tissue damage is frequently aligned with local modulations of neuronal and glial gap junction channel expression levels. The degree of gap junctional protein expression and intercellular coupling efficiency, as well as hemichannel function has substantially impact on the course of trauma recovery and outcome. During TBI, gap junctions are especially involved in the intercellular molecule trafficking on repair of blood vessels and the regulation of vasomotor tone. Furthermore, gliosis and astrocytic swelling due to mechanical strain injury point out the consequences of derailed gap junction communication. This review addresses the outstanding role of gap junction channels in TBI pathophysiology and links the current state of results to applied clinical procedures as well as perspectives in acute and long-term treatment options.

## Introduction

Traumatic brain injury (TBI) is defined as a severe intracranial injury due to external impact force. Insurance industry and many health care providers treat TBI as an “event.” Once treated with a brief period of rehabilitation, the perception exists that patients with a TBI require little further treatment and face no lasting effects on the central nervous system or other organ systems (Lu et al., 2013; Selvarajah et al., 2013). As a chronic disease TBI meets the World Health Organization definition as having one or more of the following characteristics: it is permanent, caused by non-reversible pathological alterations, requires special training of the patient for rehabilitation, and/or may require a long-period of observation, supervision, or care. TBI increases long-term mortality and reduces life expectancy. It is associated with increased incidences of seizures, sleep disorders, neurodegenerative diseases, neuroendocrine dysregulation, and psychiatric diseases that may arise and/or persist for months to years post-injury.

One of the major factors in cellular pathophysiology, leading to long-lasting effects in TBI patients, is based on the interruption of gap junction connectivity of the neuro-glial network and mechanically induced opening of large pore channel conductances due to the traumatic event (Avila et al., 2011).

**Figure 1:**
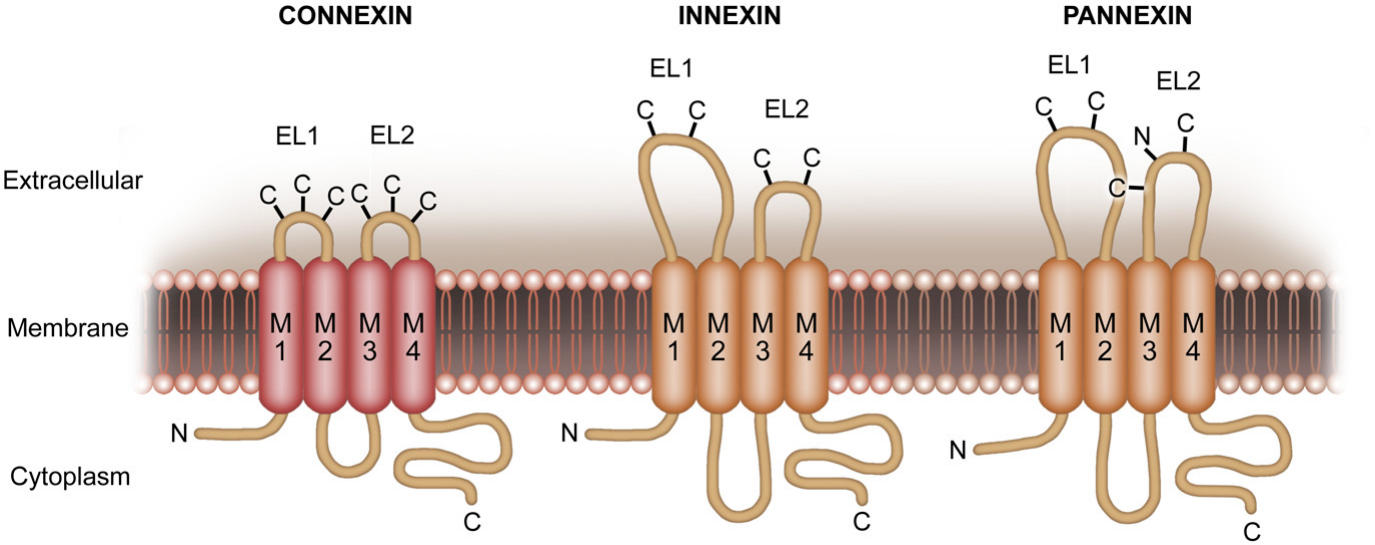
**More recently, another family of proteins, termed pannexins (Panxs), has been identified**. These proteins share similar membrane topology but no sequence homology with Cxs. In turn, sequence homology is given to the invertebrate gap junction protein Innexins. Pannexins form unique, multimeric membrane channels with pharmacosensitivity partially overlapping with that of Cx hemichannels.

Gap junctions enable neurons and glial cells to communicate by exchange of ions and small molecules via intercellularly connected, adjacent channel proteins (Prochnow and Dermietzel, 2008; Eugenin et al., 2012). Each of these channels is consisting of six subunits of integral membrane proteins, termed Connexins (Cxs) (Figure 1). In mice and humans, Connexins are encoded by a family with 20 and 21 genes, respectively (Berthoud and Beyer, 2009). In the CNS of rodents, the majority of Connexin isoforms (*n* = 11) are expressed by glial cells (Prochnow and Dermietzel, 2008; Giaume and Theis, 2010; Mika and Prochnow, 2012; Rash et al., 2012). Among others, Cx36 gives rise to the main neuronal gap junction channel forming protein (Belluardo et al., 2000; Venance et al., 2000; Rash et al., 2012; Belousov and Fontes, 2013). Connexins can also be expressed as unopposed hemichannels in cells of the CNS. In this context, different types of Cxs form hemichannels (Schock et al., 2008; Giaume and Theis, 2010; Saez et al., 2010).

Additionally, a vertebrate protein family, Pannexins (Pxs) (Figure 1), with a strong homology to Innexins (Figure 1) has been described (Panchin et al., 2000; Baranova et al., 2004; Panchin, 2005). Innexins are the gap junction forming proteins in insects. Recent findings reveal that Px1, expressed on neurons and glial cells (Thompson et al., 2008; Macvicar and Thompson, 2010; Bargiotas et al., 2011; Prochnow et al., 2012), operates as a unique large pore channel instead of forming gap junctions (Bao et al., 2004; Locovei et al., 2006a,b; Pelegrin and Surprenant, 2006, 2007; Romanov et al., 2007; Zoidl et al., 2007).

Here we discuss the role, function, and regulation of Connexin-based gap junctions and hemichannels as well as Pannexin1 large pore channels (summarized in Table 1) in the context of concomitant network (dys-)regulation and pro-inflammatory effects according to TBI. Furthermore, the latter proteins will be focused with regard to experimental and clinical treatment options which might influence the long-term outcome in TBI patients in future times.

**Table 1:** **Summary of cell type specific expression of Cxs and Pxs as described until this point of time**.

**Cell type**	**Connexin isoforms**	**Pannexin isoforms**	**Discussed by, a.o**.
Neurons	26, 30.2, **36**, 45, 57	**1**, 2	Condorelli et al., 2000
			Rouach et al., 2002
			Bruzzone et al., 2003
			Nagy et al., 2004
Astrocytes	26, 30, **43**, 40, 45, 46	1	Nagy et al., 2004
			Giaume et al., 2013
Oligodendrocytes/Myelin	**29, 32**, 47	Not described	Nagy et al., 2004
			Altevogt et al., 2002
			Li et al., 2014
Microglia	36, **43**, 45	Not described	Mika and Prochnow, 2012

Bold typed isoforms are currently known to contribute to the course and development of traumatic brain injury (a.o., and others).

## The role of gap junctions and connexin hemichannels during CNS injury

### Neuronal connexins

Electrical synapses based on Cx36/Cx35gap junctional contacts are common for dendro-dendritic as well as dendro-somatic neuronal connections and contacts between axon terminals (Hamzei-Sichani et al., 2007). In the mammalian central nervous system, Cx36 is expressed by neurons in a dynamic manner: the coupling of neurons by gap junctions and the expression of Cx36 transiently increase during early postnatal development and take part in the regulation of neuronal death (Park et al., 2011). After the second postnatal week, coupling and expression of Cx36 physiologically decrease (Arumugam et al., 2005).

Due to spinal cord and TBI, gap junctional coupling and Cx36 expression by neurons increase in a pathophysiologic way (Chang et al., 2000; Frantseva et al., 2002a). A similar injury-dependent upregulation of neuronal coupling occurs during ischemia (Oguro et al., 2001; De Pina-Benabou et al., 2005) and epilepsy (Gajda et al., 2003). Furthermore, the isoforms Cx26 and Cx32 can be expressed by neurons of the CNS (Bruzzone and Ressot, 1997; Dermietzel and Hofstadter, 1998; Teubner et al., 2001). Belousov and colleagues characterized the mechanisms that are responsible for the injury-mediated increase in neuronal gap junction coupling. Furthermore, they were able to describe a group II metabotropic glutamate receptor (mGluR) regulation dependent process in injury-induced neuronal death (Wang et al., 2012). It was shown that activation of group II mGluRs increases background levels of neuronal gap junction coupling and Cx36 expression. Inactivation of group II mGluRs prevents ischemic increase in coupling of Cx36 via cAMP/PKA-dependent signaling pathways (negatively coupled to the group II mGluRs) (Conn et al., 2005). Interestingly, other neurotransmitter receptors, including N-methyl-D-aspartate receptors (NMDAR), AMPA receptors, group I mGluRs, group III mGluRs, GABA_A_ receptors, and GABA_B_ receptors did not directly contribute to these regulatory mechanisms (Wang et al., 2012). Similar results were obtained using three other *in vitro* injury models: hypo-osmotic shock, as a model of cytotoxic and osmotic edemas occurring during stroke and TBI (Unterberg et al., 2004); hydrostatic pressure injury, representing mechanical aspects of TBI (Morrison et al., 1998a,b) and administration of 4-aminopyridine, as a model of epileptic seizures (Wong and Yamada, 2001).

These observations led to the conclusion that group II mGluRs controls the injury-mediated increase in neuronal gap junction coupling. By regulation of neuronal gap junctions, they also control both death and survival mechanisms in injured neurons.

The critical factors for secondary neuronal cell death can be restricted to an excessive glutamate release from cells of the injured tissue, in turn causing local glutamate-dependent excitotoxicity. The excitotoxic mechanisms of glutamate include hyperactivation of NMDA receptors and increased Ca^2+^ ion influx. Furthermore, an overactivation of Ca^2+^-dependent signaling pathways is discussed to cause death of neurons (Choi, 1988; Arundine and Tymianski, 2004; Hazell, 2007).

Based on the results of Belousov et al. (Belousov et al., 2012; Belousov and Fontes, 2013), a novel model for the mechanisms of glutamate-dependent excitotoxicity was proposed. This model implicates that overactivation of NMDAR is not only the main reason for glutamate-dependent neuronal death during neuronal injury. The process might be supported by the expression of neuronal gap junctions.

### Astroglial connexins

TBI is mostly associated with excessive excitatory neurotransmitter release and subsequent cellular depolarization. This process, in turn, impacts on the degree of neuronal injury and glial reaction. The abrupt increase of extracellular glutamate subsequently drives an influx of Ca^2+^ ions, resulting into Ca^2+^-dependent excitotoxicity (Conti et al., 1998; Osteen et al., 2001). Another face of TBI is the rapid loss of astrocytes and the substitutive induction of reactive astrocytes, as it could be demonstrated in the Hippocampus in animal models (Dietrich et al., 1994; Zhao et al., 2003). Studies of Nakase et al. (2003) gave rise for a predominant role of astroglial Cx43-gap junctions in the reduction of neuronal apoptosis under ischemic conditions, influencing infarct volumes and caspase-3 levels. Furthermore, the physiologically relevant portion of glial fibrillary acidic protein (GFAP)—positive astrocytes could be primarily attributed to Cx43 expressing mice. Knockout models showed that a lack of Cx43 expression is aligned with strongly reduced levels in GFAP expression, leading to pathophysiological conditions of the neuro-glial syncythium (Nakase et al., 2003). Cx43 and Cx30 are primarily localized on astrocytes, whereas Cx29, 32, and 47 are predominantly expressed on oligodendrocytes (Bruzzone and Ressot, 1997; Dermietzel and Hofstadter, 1998; Teubner et al., 2001).

Regarding functionality, the intracellular condition and state of phosphorylation of the Connexin proteins influence the intercellular permeability characteristics of gap junctions. In this context it was demonstrated that extracellular signal-regulated kinase (ERK) mediated phosphorylation of Cx43 strongly reduced the intercellular permeability of gap junctions (Brandes et al., 2002; Cottrell et al., 2003). These findings correlate well with later findings of Ohsumi et al. (2010) who investigated the expression of phosphorylated Cx43 in the rat hippocampus following experimental TBI *in vivo*. The authors showed by the use of immunochemistry that high levels of phosphorylated Cx43 were present in astrocytes of the hippocampal CA3 of the TBI exposed hemisphere for up to 6 h. Subsequent upregulation of phosphorylated ERK coincided with the Cx43 expression and gave rise to a participation of astrocytic gap junctions in the hippocampal pathophysiology following TBI (Ohsumi et al., 2010).

It is critically discussed whether propagation of stress factors (including apoptotic factors) via astroglial gap junctions might impact on the process of secondary brain damage after TBI which is mostly correlated to ischemic injury (Hossmann, 1994; Lin et al., 1998; Frantseva et al., 2002a). This hypothesis became strengthened by findings of Frantseva and colleagues who demonstrated that neuronal cell death after hypoxic-hypoglycemic insults is reduced in CNS slices treated with antisense oligonucleotides for Cx43. Propagation of secondary cell injury to neighbored cells via astrocytic gap junctions was demonstrated in 1998. It was shown that astrocytic Cx43 based gap junctions mediate transcellular signals, enhancing calcium overload, oxidative stress, and metabolic inhibition (Lin et al., 1998). On the other hand Nakase et al. (2003) suggested that astrocytic gap junctions facilitate the establishment of a reactive astrocytic network and remove cytotoxic factors (Nakase et al., 2003). This would also underline the indication that mechanisms of spreading depression after TBI or ischemic insults trigger neuronal as well as glial depolarization, and thereafter tend to induce neuronal damage (Katayama et al., 1990; Nedergaard and Hansen, 1993).

Especially in the context of cerebral ischemia, selective blockade of Cx43 hemichannels by mimetic peptide application significantly improved the neuronal outcome in fetal sheep. This finding was in particular due to a hemichannel blockade following, but not during global cerebral ischemia in the near-term fetal condition (Davidson et al., 2013). Recently, Orellana et al. (2013) discussed the role of Cx hemichannels as diffusional pathways for ions and small molecules between the intra- and extracellular compartments and suggest that channel opening may gate the release of neurotoxic substances (e.g., glutamate, external Ca^2+^-overload) (Orellana et al., 2011). Here, putative operators, facilitating channel activity can be cytokines such as Tumor Necrosis Factor-alpha or Interleukin1-β (Froger et al., 2010). In other cases, hemichannels may confer neuroprotection against an ischemic episode by the phenomenon of ischemic preconditioning (Bennett et al., 2012).

Nevertheless, it can be summarized to this point that the cell type specific expression of neuronal and astroglial Cxs seems to be dynamic and influenced by the environmental signals that are induced by TBI. The role of neuronal and astroglial Cxs in neuronal cell death as well as their role in neuroprotection increase in therapeutic importance.

### Pannexins and TBI

Addressing Pannexin functions, especially in the context of neurotrauma, we enter a young field in CNS research. In 2003, three Innexin homologs were cloned from rats and mice, the isoforms Px1, Px2, and Px3. Since nothing is known about the role of the latter two isoforms in TBI, this paragraph focusses on the sparse facts about Px1 in neuroinflammation and induced neurotrauma-like conditions. Pannexin1 is ubiquitously expressed in the mammalian CNS (Bruzzone et al., 2003; Ray et al., 2005), covering cells of the retina, olfactory bulb, neocortex, hippocampus, cerebellum, and spinal cord. Pannexin1 is described to act as an ATP release channel with unselective high conductance properties (Prochnow et al., 2009; Kurtenbach et al., 2013). Opening of this large pore channel can be initiated by depolarizing voltage sensing or hyposmolar/mechanical activation (Iglesias and Spray, 2012; Krizaj et al., 2013). Regarding traumatic injury of the CNS, the mechanical stress related activation process of Panx1 gains special interest. With focus on inflammatory processes, neuronal Px1 was shown to closely operate together with stimulus-induced opening of astroglial Cx43 hemichannels with. According to this, pharmacologic inhibition of NMDA or P2X receptors only partially reduced the activation of neuronal Panx1 hemichannels and neuronal mortality (Orellana et al., 2011). Simultaneous inhibition of both receptors completely prevented the neurotoxic response. The authors suggested that responses to ATP and glutamate converge in activation of neuronal Panx1 hemichannels. It was proposed that blocking hemichannels expressed by astrocytes and/or neurons in the inflamed nervous system could represent a novel and alternative strategy to reduce neuronal loss in various pathological states including ischemia which often concomitantly occurs as a consequence of TBI.

## Experimental models elucidating the function of connexins and pannexins in TBI

Sudden cumulation of extracellular glutamate after TBI results into an increase of intracellular Ca^2+^ ion which, in turn, leads to Ca^2+^-dependent excitotxicity (Conti et al., 1998; Osteen et al., 2001). Animal models reveal that vulnerability of CNS structures does not follow a homogenous propagation pattern, in contrast, selective vulnerability is observed for certain regions such as CA3 pyramidal neurons, dentate hilar neurons, and cortical neurons (Lowenstein et al., 1992; Cortez et al., 1995; Nawashiro et al., 1995). These results were primarily obtained from fluid percussion induced neurotraumatization (McIntosh et al., 1989) in rats. In this experimental setting, focal brain injury was performed by application of fluid pulse (2.6–2.8 atm; 12 ms duration) on the intact dura following osteotomy in the anaesthetized animal. By this approach, Ohsumi et al. investigated the temporal and spatial expression profiles of Cx43 in the hippocampal CA3 region of adult rats (Ohsumi et al., 2010). Using Western blot analysis and immunohistochemistry the authors were able to show that phosphorylated Cx43 is strongly increased in astrocytes of the perilesion sites 1 h after TBI and lasting for the next 24 h. In contrast, no distinct pattern of phosphorylated Cx43 expression was found in the neighbored hippocampal CA1 region. Immunoreactivity of the total amount of Cx43 was unchanged. Based on these facts, the authors suggested that time-dependent phosphorylation of Cx43 contributes to hippocampal dysfunction via astrocytic gap junction communication and serves as a prodromal. Total Cx43 immunoreactivity was localized in astrocytes in the direct periphery of the contusion focus at 72 h post TBI. This probably directly reflects the anatomical correlate for potentiation of intercellular signaling via gap junction transduction. This process might be beneficial to restoring of neuronal damage during the late phase of injury.

In analogy to an increased occurrence in war zones and due to terrorist incidents (Risdall and Menon, 2011), another set of approaches covers blast-induced traumatic brain injuries in animal models (Wolf et al., 2009). Also in this type of experimental setting, utilizing controlled 60 mg TNT blasts in a described vicinity of rabbit skulls, an increase in Cx43 and caspase-3 expression could by detected in the penumbral regions. Caspase-3 is one of the downstream pro-apoptotic factors, discussed as a candidate to be transmitted via gap junction communication during TBI (Wennersten et al., 2003; Yong-Ming et al., 2012).

Providing a target for putative treatment the penumbra region of TBI delineates, as a locus for delayed responses, the primary injury core from the intact brain region (Paciaroni et al., 2009). The region itself reveals a centralized pattern of astroglial Cx43 expression which is described to be related to ischemic processes (Hossain et al., 1994). Later on it was demonstrated that Cx29 and Cx32 are expressed in a subset of microglia and astrocytes in the sharp border of the penumbra (Moon et al., 2010). The data were obtained from a cryogenic TBI model in mice where a liquid nitrogen filled iron rod is directly placed on the cranium of the anaesthetized animal (Tatsumi et al., 2005). The authors suggested a Connexin-mediated influence on cellular degeneration/regeneration information between the core and the periphery of the injury. This finding was also underlined by knockout animal studies. As an example, knockout of the Cx32 gene enhanced neuronal vulnerability (Oguro et al., 2001; Moon et al., 2010), whereas knockout of the Cx43 gene resulted in the previously described decreased neuronal vulnerability after TBI (Frantseva et al., 2002a).

Regarding the investigation of Pannexin channel function due to neurotrauma the only reliable model are specified weight drop experiments by Wang et al., on the spinal cord in mice (Wang et al., 2004). In the weight drop approach, a standardized neurotrauma is induced by dropping a small weight into a predefined area of the spinal cord. By help of this attempt, Wang and colleagues showed that ATP was released in the penumbral region leading to a massive recruitment of microglial, macrophage, and monocyte derived cells. The process was followed by secondary expansion of the lesion (Wang et al., 2004). The authors were also able to identify the contribution of P2X receptor activation and neuronal excitation in direct subsequence to the lesion process. Evidence was provided by targeting direct pharmacological inhibition of ATP-P2X7 receptor interaction and application of P2X7 receptor antagonists. In combination with previous findings of Nedergaard and colleagues (Wang et al., 2004; Peng et al., 2009) these results gave first rise for an involvement of Px1 interaction with P2X7 receptors in the course of neurotrauma.

Another sophisticated approach was given by Davalos et al. (2005). The authors investigated the fast microglial response according to local two-photon laser mediated ablation of the superficial layer of the motor cortex and mechanical tissue manipulation via a glass microelectrode. Wang et al., as well as Davalos et al., reported and mimicked the effects of locally released ATP. Davalos and colleagues showed that local increases in ATP levels, as it can be supported by Px1 operated release from astrocytes and neurons, influence the chemotactic guidance of microglial processes toward the borders of the penumbral region in mice *in vivo*. The process is carried out without movement of the cells' somata and can be mimicked by local application of ATP and subsequent activation of P2Y receptors. Additionally the baseline motility of microglial processes was slowed down under application of ATP-degrading apyrase and Connexin channel inhibitors, indicating that the interplay of both, Connexins and Pannexins seems to be needful in the cellular response toward injury. Nevertheless, the different types of experiments underline that severity and type of traumatization can impact on Px1- and Cx-dependent systems in relevant modulatory ways with influence on the future development of the injury site.

## From experiments to bedside? a young chapter

Based on the actual results from *in vitro* and *in vivo* related experiments, Connexins appear to be the most important player in the intercellular neuroglial pathosphysiology of the CNS during TBI. At the moment, an effective therapeutic agent for TBI remains elusive (Wang et al., 2006; Beauchamp et al., 2008; Elliott et al., 2011).

The astroglial expression site of Cx43 (Garre et al., 2010) might play an important role in future clinical treatment. Since description is pending that stress factors, including apoptotic factors, are passed through gap junctions, the hypothesis was established that a partial decrease in neuronal or glial gap junction mediated communication might be sufficient to reduce secondary tissue damage. The hypothesis became strengthened by studies of Frantseva et al. who proved that cell death is reduced in native CNS slices following treatment with antisense oligonucleotides for Cx43 after hypoxic-glycemic insult (Frantseva et al., 2002b). Pharmacological inhibition of Cx43 activity was performed in a multitude of TBI studies. Here, gap junction blockers such as insulin (Homma et al., 1998) octanol (Rawanduzy et al., 1997) and carbenoxolone (De Pina-Benabou et al., 2005) were shown to exhibit neuroprotective effects in diverse models of cerebral injury. Especially the latter one is also serving as a non-specific Px1-inhibitor.

Stem cell therapy promises another treatment option of TBI. In order to reestablish neuronal networks in the perilesion regions, the functional integration of grafted neuronal stem cells might provide a perspective. In this context the property of Cx43 based cellular coupling should serve as a means to link endogenous and exogenous network components together. Aside from the expression on astrocytes, Cx43 is most closely associated with immature neuronal stem cells (Jaderstad et al., 2010; Yu et al., 2013). One proposed way in which grafted neuronal stem cells integrate into host tissues is gap junction coupling. The exogenous stem cells are described to protect the host neurons from cell death as well as reducing signs of secondary injury such as reactive gliosis. Experiments *in vivo* and *in vitro* revealed that these beneficial effects were lacking, when gap junction communication was suppressed in the host (Jaderstad et al., 2010). Yu et al. (2013) underlined these findings by showing that neuronal stem cell transplantation significantly enhanced the Cx43 protein expression in a rat model of controlled cortical impact TBI (Ma et al., 2011). Connexin43 expression after transplantation was increased in the core and the border of injury. These expression levels were maintained for up to 4 weeks post transplantation, giving rise that gap junctions based on Cx43 might participate in the iterative developmental process of graft integration into the host CNS tissue following TBI. Although it is becoming recognized that grafted neuronal stem cells interact with endogenous neurons of the peritransplant region, the underlying mechanisms remain to be matter to intensified research.

Since approximately 85–89% of TBI patients show non-open trauma injuries e.g., caused by traffic accidents (Masson et al., 2001; Wu et al., 2008), local pharmacological treatment or stem cell delivery to the host tissue still provides a problem.

An actual pharmacological intersection between basic research and applied treatment of TBI is given by the patient administration of cannabinoid receptor agonists including Δ^9^-tetrahydrocannabinol or nabilone. These substances are clinically used in the suppression of multiple sclerosis and spinal cord injury symptoms, as well as for the prevention of neurotoxicity (Pertwee, 2006). Interestingly, cannabinoid receptor agonists influence Cx43 regulation in culture models under pro-inflammatory conditions in a preventive manner mediated by CB1 receptor activation (Froger et al., 2009). On the one hand Froger et al. demonstrated that cannabinoid agonists prevent the inhibition of gap junction coupling of astrocytes induced by conditioned medium harvested from LPS-activated microglia. On the other hand the authors showed via dye coupling and ethidium uptake experiments in astrocyte cultures the dual regulation of Cx43 functions induced by IL-1β and TNF-α.

These results of cannabinoid-Cx interaction provide evidence for an indirect mechanism to impact on Cx43 activity in a traumatic/pro-inflammatory condition as it might act already in clinical routine.

### Conflict of interest statement

The author declares that the research was conducted in the absence of any commercial or financial relationships that could be construed as a potential conflict of interest.
